# Intensified hand-hygiene campaign including soap-and-water wash may prevent acute infections in office workers, as shown by a recognized-exposure -adjusted analysis of a randomized trial

**DOI:** 10.1186/s12879-016-2157-z

**Published:** 2017-01-09

**Authors:** Tapani Hovi, Jukka Ollgren, Carita Savolainen-Kopra

**Affiliations:** Department of Infectious Diseases, Viral Infections Unit, National Institute for Health and Welfare (THL), P.O. Box 30, FIN-00271 Helsinki, Finland

**Keywords:** Respiratory infections, Gastrointestinal infections, Office work, Hand-hygiene, Cluster-randomized trial, Self-reported data, Exposure to infected persons, Longitudinal data, Seasonality, Bayesian analysis methods

## Abstract

**Background:**

Variable exposure to causative agents of acute respiratory (RTI) or gastrointestinal tract infections (GTI) is a significant confounding factor in the analysis of the efficacy of interventions concerning these infections. We had an exceptional opportunity to reanalyze a previously published dataset from a trial assessing the effect of enhanced hand hygiene on the occurrence of RTI or GTI in adults, after adjustment for reported exposure and other covariates.

**Methods:**

Twenty-one working units (designated clusters) each including at least 50 office employees, totaling 1,270 persons, were randomized into two intervention arms (either using water-and-soap or alcohol-rub in hand cleansing), or in the control arm. Self-reported data was collected through weekly emails and included own symptoms of RTI or GTI, and exposures to other persons with similar symptoms. Differences in the weekly occurrences of RTI and GTI symptoms between the arms were analyzed using multilevel binary regression model with log link with personal and cluster specific random effects, self-reported exposure to homologous disease, randomization triplet, and seasonality as covariates in the Bayesian framework.

**Results:**

Over the 16 months duration of the trial, 297 persons in the soap and water arm, 238 persons in the alcohol-based hand rub arm, and 230 controls sent reports. The arms were similar in age distribution and gender ratios. A temporally-associated reported exposure strongly increased the risk of both types of infection in all trial arms. Persons in the soap-and-water arm reported a significantly – about 24% lower weekly prevalence of GTI than the controls whether they had observed an exposure or not during the preceding week, while for RTI, this intervention reduced the prevalence only during weeks without a reported exposure. Alcohol-rub did not affect the symptom prevalence.

**Conclusions:**

We conclude that while frequent and careful hand washing with soap and water partially protected office-working adults from GTI, the effect on RTI was only marginal in this study. Potential reasons for this difference include partially different transmission routes and a difference in the virus load. In this trial, frequent standardized hand rubbing with ethanol-based disinfectant did not reduce the weekly prevalence of either type of infections.

**Trial registration:**

ClinicalTrials.gov Identifier: NCT00821509, 12 March 2009.

**Electronic supplementary material:**

The online version of this article (doi:10.1186/s12879-016-2157-z) contains supplementary material, which is available to authorized users.

## Background

Enhanced hand hygiene is a well-established means to prevent transmission of infections in hospital settings [1] as well as in other semi-closed environments with high infection pressure, such as day care centres [2–5], schools [6, 7] and military service [8]. In open community settings, the evidence for efficacy has been considered inconclusive [9, 10], and both positive effects [11] and lack of efficacy [12] have been reported in more recent studies. This divergence of the published results may be partly due to the multitude of difficult to control intervention-unrelated factors potentially affecting the outcome of the trial [13], which may result in a failure to identify all relevant factors that could be used in pre-randomization stratification of the population in order to improve arm matching. In addition, post-randomization emergence of unexpected confounding factors may complicate the analysis of the results.

In the 16 months STOPFLU Study, a cluster-randomized intervention trial some time ago [13], we tried to harmonize the study arms for potential exposure to other infected individuals by calculating a designated infection risk sum for each of the study clusters, to be used in the randomization. The intervention arms in this occupational health study included behavioral changes aiming at limitation of the transmission of infectious agents, and two different hand cleansing methods. The originally planned primary outcome was the rate of self-reported acute respiratory (RTI) and gastrointestinal tract infections (GTI) in the study clusters [13]. As discussed before [14], the performance of the trial was challenged by unexpected business-operational problems in some of the six committed corporations, unknown amount of “leakage” or contamination of the control clusters, and moreover, emergence of an outbreak of the A/H1N1v influenza pandemic in Finland. The latter resulted in a well-publicized national campaign including recommendations for enhanced hand hygiene in order to minimize the disease burden due to the pandemic. As a consequence, the setup of our study was somewhat compromised as the controls were naturally included among the targets of this nationwide campaign. Because of these undesired changes in the study setup, we initially made a simple global comparison of the infection rates of self-reported infection episodes between the arms before and during the pandemic and concluded, that frequent washing of hands with soap and water may reduce the rate of acute infections under the conditions used [14].

Later on we had the opportunity to analyze another set of data collected from the participants of the STOPFLU Study, the self-reported exposures to other people with obvious signs of RTI or GTI. We found that self-reported homologous exposures remarkably increased the relative risk of both self-reported RTI and GTI in this trial [15]. Therefore, we decided to reanalyze the main outcome of the STOPFLU Study with self-reported exposure, successive events, seasonality, cluster effects, and randomization triplet included in the statistical model as covariates. Different statistical models were tested, including sensitivity analyses with selected variables.

## Methods

### Study design

We studied the efficacy of enhanced hand hygiene on the occurrence of RTI and GTI symptoms in office environment in an open, cluster-randomized intervention trial. A detailed description of the study design has been reported earlier [13]. In short, a total of 21 distinct office work units, later referred to as clusters, were identified in six corporations in the Helsinki Region. In collaboration with the staffs of the occupational health clinics of the corporations, volunteers were recruited among the 1,270 employees working in these units, after excluding persons with open wounds or chronic eczema in hands. This group received a personal email from the researchers, including an electronic contagion risk survey enquiring about e.g. type of children’s day-care, potential smoking, frequency of work trips, physician diagnosed chronic diseases, etc. [13]. An arbitrary virus transmission risk score was calculated for each cluster based on the results of the contagion risk survey. The clusters were then ranked according to the score and divided in seven triplets on the basis of the rank [14]. One member of each triplet was finally randomized into each of the three trial arms. According to the protocol, all new employees to be hired into these work units during the trial had to be offered a possibility to participate in the study. The participants had the right to stop reporting temporarily during holidays and also completely, without giving a reason. As a result, it was clear from the beginning that the individual follow-up times will show a range of variation between the participants. For these reasons, the planned initial outcome was the number of RTI or GTI episodes (defined below) in a given cluster over the sum of reported person-weeks in the cluster [13].

The trial arms were: 1) the soap and water wash arm (later, ‘soap-and-water arm’); 2), the alcohol-based hand rub arm (‘alcohol-rub arm’); and 3) the control arm. Toilets at the workplaces were equipped with liquid hand soap (all arms) and, in the alcohol-rub arm, with alcohol-based hand rub. Participants in the intervention arms also received bottles of the arm-specific hand hygiene product to be used at home and, in the case of alcohol hand rub, personally in the office. In addition to personal guidance in hand cleansing specific to each arm, the participants of the two intervention arms received guidance on how to otherwise limit the transmission of infections, e.g. coughing, sneezing into a disposable handkerchief or alternatively the sleeve, and avoiding shaking hands [13]. Participants of the control arm did not receive any specific guidance regarding hand hygiene or behavioral instructions for limiting transmission of infections, but of course, they also received relevant background information during the recruitment. The aim was to compare the two intervention arms with the control arm separately, rather than between each other. The interventions were not blinded to any party involved (i.e. the study group, participants or the occupational health services).

### Data collection and processing

Symptoms typical of acute RTI and those of GTI were described in detail during the in advance training of the participants and repeatedly in the weekly emails. Daily infection symptoms were recorded by the participants in a weekly self-report using an internet-based questionnaire, a link to which was sent via e-mail [13]. The questionnaire also requested giving information on possible exposure to other persons with signs of RTI or GTI during the report week, as well as whether the possible exposure had been experienced at work, or outside work, or both. An exposure was defined as “having met people with obvious symptoms of either respiratory or gastrointestinal infection”, without stating in writing relevant physical distance or minimal time of a designated contact, or the number of assumed contact events during a given report week. The software used for data collection was acquired from Digium Enterprises, Espoo, Finland. An overview of the progress of the trial is shown in the standard flowchart, Additional file 1.

In the initial analysis [14], the reported occurrence of symptoms of RTI or GTI in the reporter was converted to designated RTI or GTI episodes (or “infection episodes” without specifying the type of symptoms), each including successive days with reported symptoms of RTI or GTI, respectively, and allowing coincidence of the two separately recorded types of episodes. In the current trial, however, because of the inclusion of the reported exposure as a covariant, and because of our current knowledge of the short median lengths of the reported infection episodes [16] we converted the daily data to “weeks with reported symptoms of RTI or GTI”, or more briefly weeks with RTI or GTI, respectively, accepting the possibility that some designated episodes may be scored twice in this way. We then assessed the association of individual weeks with reported exposure to persons with RTI and/or GTI, with self-reported occurrence of homologous symptoms during the same or the following week. A relative risk (ReR) of exposure-associated disease was calculated for the “same week” data using the following formula:

$$ ReR=\frac{\mathrm{WE}+}{\mathrm{WE}-} $$

where WE+ stands for the proportion of weeks with reported homologous exposure out of all weeks with reported symptoms, and WE- for the proportion of weeks without a reported homologous exposure out of all weeks with reported RTI symptoms. The corresponding ReR for the “following week” data was calculated similarly but taking in account a possible reported homologous exposure during the preceding, rather than the coinciding week. The relative risk associated with reported exposure thus indicates how much more likely symptoms of disease would emerge during the indicated week if a homologous exposure has been recognized. -A risk ratio (RRa) for the intervention arms was calculated by dividing the ReR of the arm in question with that of the control arm, and reflects the efficacy of the intervention. The RRas were calculated separately for weeks associated with a homologous exposure and for those without a reported temporal coincidence.

When designing this trial, the research group anticipated that, for various reasons, all participants will not adhere to weekly reporting throughout the expected more than 1 year duration of the trial. According to the protocol the main endpoint was the cumulative number of infection episodes in a given study cluster over the cumulative number of reported follow-up weeks in the cluster. Therefore, individual ceasing of reporting should not be considered as a conventional drop out case in the data analysis.

### Statistical analysis

Our main aim was to estimate the effects of intervention arms to weekly prevalence of RTI and GTI symptoms, and possible interaction of the arms with reported exposures during the same or the preceding week.. Accordingly, we calculated (posterior) predictive margins of the prevalence of weekly reported symptoms in the arms and the relative risks between the intervention arms with and without a reported exposure. To model the effects of the explanatory variables in our cluster randomized longitudinal study, hierarchical mixed effect models were fitted to the data. The outcomes in the models were weekly reported symptoms for RTI or GTI. The weekly prevalence of those were modelled using a binary regression model with log link. The clustering effects were taken into account by normally distributed random effects at log-scale. Due to the unbalanced individual reporting in the study the longitudinal effects were modelled simply by the random effect term i.e. heterogeneity. The results were also seasonally adjusted using the calendar month as a fixed effect. We also included the trial design matching into triplets in the model in different ways: by random/fixed effects and by using a matching score and inspecting the sensitivity of the results. The calculations were done in Bayesian framework [17] using Markov chain Monte Carlo (MCMC) -methods and Winbugs 1.4.3 –program (Imperial College & MRC, UK). As many as 200,000 to 500,000 iterations were needed for ensuring the convergence. We found that the effect of matching was negligible to estimates and variances (posteriors) and in the final calculations we dropped out the triplet effect. All the priors used in modelling were non-informative.

In order to estimate the arm effect after adjusting for the possibly different exposure pressure in different clusters, we adjusted for the reported exposure so that only the unreported, possibly unrecognized part of exposure remained unadjusted. We also included interactions between the reported exposure and the arms in the regression model. However, the adjustment with the reported exposures may have caused a bias, due to over-adjustment [18], in the estimation of the arm effects and the interactions because of the following: The expected effects of the interventions on the prevalence of the symptoms were through an assumed reduction of the infection exposure pressure. In the regression model we used descending proxies of the true prevalence of both the adjusting variable and the outcome, i.e. reported exposures and reported weeks with symptoms, respectively. This is likely to cause an attenuation of the intervention effect to the outcome. Overall, the intervention arm effects after the adjustments were almost the same as without adjusting for the reported exposure but the estimated interaction effects showed that the interventions were less effective in preventing the appearance of symptoms associated with reported exposures.

We also conducted some sensitivity analyses using marginal models (generalized estimating equation, GEE) as described in the Additional file 2.

## Results and discussion

### General aspects

Recruitment, drop-outs, new recruiting, and reporting coverage have been described earlier in detail [14]. Briefly, the recruitment took place in January and February 2009. Altogether 683 persons out of 1,270 eligible employees initially volunteered to participate in the study. Subsequent drop-outs and new recruitments are shown in the flowchart (Additional file 2). Finally, data from 297 persons in the soap-and-water arm, from 238 persons in the alcohol-rub arm, and from 230 controls were available for analysis (Table 1). As reported before [14], the mean and median ages and the age ranges were similar in the three arms. The proportion of females appeared marginally higher in the controls than in the two intervention arms (Table 1). Seventy six percent of volunteers who started reporting continued to do so until the end of May 2010 when the study ended. The median weekly reporting coverage remained high throughout the trial and was similar in all trial arms [14]. None of the participants reported harmful effects.

**Table 1:** Demographic data of the healthy volunteers in the trial

Trial arm^a^	Number of reporting participants		Gender ^b^		Age (years)	
	Initially	Finally^c^	Females	Males	Mean (SD^d^)	Range
Water and soap	257	297	202	80	45.1 (10.2)	22 – 64
Alcohol hand rub	202	238	170	53	42.7 (10.1)	20 - 63
Control	224	230	173	61	42.9 (11.1)	21 – 62

^a^Participants in the two intervention arms received instructions for transmission-limiting behavior when sneezing or coughing and for frequent hand cleansing with the indicated method

^b^A few participants did not give this information (not obligatory according to the protocol)

^c^Total number of participants providing data for one or more follow-up week (including those recruited after the onset of the trial)

^d^SD, standard deviation

Altogether 38,644 weekly reports were received. As reported before [14], in the arm instructed to use soap and water in hand cleansing the incidence of designated infection episodes, either RTI and/or GTI, was 7% lower than in the controls through all the study period, and 17% lower if only the time before the pandemic influenza outbreak was included in the analysis., In contrast, the use of alcohol-rub did not decrease the incidence of episodes. In the current study, we analyzed the data by comparing between arms the weeks with reported RTI or GTI symptoms, as well as those with reported exposures. In all arms, the proportion of person weeks with reported RTI (11.0 – 12.9%) or GTI symptoms (1.9 – 2.7) was, as expected, higher than that with an onset of the corresponding disease episode. This is because some of the designated disease episodes were registered for two successive report weeks. The proportions of weeks with reported RTI symptoms, and of those with reported GTI symptoms, were lower in the arm using soap and water than in the controls (probabilities of 0.94 and 0.96, respectively). The figures in the alcohol-rub arm were similar to those of the controls (Table 2). This result corroborates our earlier report based on straightforward R-statistics [14].

**Table 2:** Weekly prevalence of reported symptoms of respiratory (RTI) or gastrointestinal tract infections (GTI) infections, and distribution of designated disease episodes in the three trial arms

Type of infection and arm	Number of distinct infection episodes and proportion of weeks with onset^b^	Weekly prevalence of reported symptoms			
		Number and proportion (out of all follow-up weeks^a^)	Predictive margin (and 95% credible interval)	Risk ratio (RRa)^c^(and 95% credible interval)	Probability that RRa is under 1.0
RTI					
Control	974/0.084	1470/0.126	0.130 (0.114, 0.151)	NR	
Soap and water	1187/0.079	1646/0.110	0.108 (0.096, 0.122)	0.836 (0.658, 1.031)	0.954
Alcohol rub	1055/0.088	1546/0.129	0.124 (0.109, 0.144)	0.960 (0.755, 1.197)	0.639
GTI					
Control	233/0.020	313/0.027	0.025 (0.020, 0.032)	NR	
Soap and water	231/0.015	282/0.019	0,019 (0.015, 0.023)	0.740 (0.510, 1.049)	0.960
Alcohol rub	229/0.019	274/0.023	0.023 (0.018, 0.029)	0.902 (0.620, 1.1281)	0.720

*NR* not relevant

^a^Total numbers of reported person weeks were 11,644, 15,014, and 11,986 in the control, soap and water, and alcohol rub arms, respectively

^b^Data from reference [14]

^c^RRa = risk ratio, ratio of the predictive margin to that of the control arm

As many as 10,817 weekly reports out of the total of 38,644 included a self-observed exposure to RTI and 2510 that to GTI. These figures are more than twofold compared to the corresponding weeks with reported symptoms of RTI or GTI. The reported exposures to both types of infection appeared to be slightly less frequent in the soap-and-water arm than in the two other arms (Table 3). In line with our previous report on the controls [15], somewhat more exposures were reported, in all trial arms, to have occurred only outside the workplace than only at work. For GTI the only-elsewhere percentages ranged from 52.6 to 57.4%, and those for RTI from 44.2 to 47.7% (Table 3). Note that person weeks with reported exposure both at work and elsewhere were a minority for both types of infection in all study arms. As the site of exposure in the control arm did not significantly affect the relative risk of the temporally associated infection [15], this variable is not included in the current reanalysis of the efficacy of the interventions.

**Table 3:** Person weeks with reported exposures to persons with assumed symptoms of respiratory (RTI) or gastrointestinal tract infections (GTI^)a^

Type of infection and arm	Weeks with reported exposure		Reported site of exposure (number of weeks and proportion out of all reported weeks with exposure)		
	Number and proportion (out of all follow-up weeks^a^)	95% credible interval	Only at work	Only elsewhere	Both at work and elsewhere
RTI					
Control	3279/0.282	0.271, 0.293	1068/0.326	1448/0.442	763/0.232
Soap and water	4031/0.268	0.259, 0.278	1308/0.324	1826/0.453	897/0.233
Alcohol rub	3507/0.293	0.282, 0.304	882/0.251	1672/0.477	953/0.272
GTI					
Control	767/0.066	0.060, 0.072	261/0.340	437/0.570	69/0.090
Soap and water	937/0.062	0.057, 0.068	313/0.334	493/0.526	131/0.140
Alcohol rub	806/0.067	0.061, 0.073	251/0.311	463/0.574	92/0.115

^a^Total numbers of reported person weeks were 11,644, 15,014, and 11,986 in the control, soap and water, and alcohol rub arms, respectively

### Effect of interventions on weekly prevalence of reported symptoms of respiratory infection

As a reported exposure to persons with assumed RTI symptoms was found to strongly increase the relative risk of occurrence of reported homologous symptoms in the controls of this trial [15] we decided to analyze possible effects of the reported exposures on the apparent effects of the two interventions. The relative risks of disease during weeks temporally associated with a reported exposure were more than four-fold during the same week and somewhat lower (about 2.5) during the following week in all trial arms. The precise numbers slightly varied but were similar with all statistical models tested (Additional file 2). Interestingly, the water-and-soap intervention did not decrease the prevalence of RTI symptoms if a homologous exposure had been reported for the same week. In contrast, weeks with RTI symptoms without a coinciding homologous exposure were significantly less frequent in this trial arm (Table 4). Symptoms occurring during a week immediately following an index week were less frequent in this arm than in the controls (probability 0.94) irrespective of a reported exposure during the index week. The figures in the alcohol-rub arm did not differ from those of the control arm (Table 4). This pattern of results was repeated with several different statistical models (Additional file 2).

**Table 4:** Reported-exposure –dependent efficacy of interventions on weekly prevalence of reported symptoms of respiratory tract infection (RTI)

Weeks with or without reported exposure Arm and number of weeks	Symptoms reported for					
	Same week			Following week		
	Number and proportion^a^	Predictive margin (CI^b^)	Risk ratio^c^ (CI^b^)	Number and proportion	Predictive margin (CI)	Risk ratio (CI^b^)
Exposure reported						
Control *n* = 3279	976/0.298	0.292 (0.258, 0.329)	NR	805/0.246	0.216 (0.192, 0.246)	NR
Soap-and-water *n* = 4031	1151/0.286	0.306 (0.276, 0.339)	1.050^d^ (0.870, 1.260)	848/0.210	0.188 (0.168, 0.208)	0.868^e^ (0.709, 1.038)
Alcohol rub *n* = 3507	1042/0.297	0.281 (0.245, 0.317)	0.937^f^ (0.771, 1.145)	838/0.239	0.207 (0.185, 0.235)	0.962^g^ (0.790, 1.16)
Exposure not reported						
Control *n *= 8365	494/0.059	0.063 (0.055, 0.072)	NR	594/0.071	0.084 (0.074, 0.096)	NR
Soap-and-water *n* = 10983	495/0.045	0.042 (0.037, 0.048)	0.678^h^ (0.546, 0.823)	707/0.064	0.073 (0.065, 0.081)	0.868^i^ (0.709, 1.038)
Alcohol rub *n* = 8476	504/0.059	0.062 (0.054, 0.071)	0.986^j^ (0.803, 1.211)	627/0.074	0.081 (0.072, 0.091)	0.962^k^ (0.790, 1.161)

*NR* not relevant

^a^Number of weeks with reported RTI symptoms representing indicated proportion out of weeks with reported exposure

^b^CI, 95% credible interval of predictive margins

^c^Risk ratio is the ratio of predictive margin in the intervention arm to that of the control arm

^defghijk^Probabilities that the risk ratio is less than 1.0; ^d^, 0.30; ^e^, 0.94; ^f^, 0.75; ^g^, 0.66; ^h^, 1.0; ^i^, 0.94; ^j^, 0.55; ^k^, 0.66

Note! e = i and g = k; no statistically significant interaction was found in the following week data between arm and exposure. Thus, a single estimate was calculated for the entire arm

It is interesting that the efficacy of the water-and-soap intervention was clearly better in the absence of reported exposure during the same week. In the control arm, about one quarter of the reported weeks with RTI symptoms occurred during person weeks without a reported exposure during the same or the preceding week [15] reflecting the long known role of subclinical infections in the transmission of respiratory infections. We do not know but we believe that under these situations, the persons may have been exposed to much lesser amounts of respiratory pathogens – which were more easily washed off - than during weeks with reported exposure possibly also including several exposure events. In addition, there might have been differences in the route of transmission. The unrecognized, washing-susceptible exposure category might be largely based on fomite and hand-mediated transmission while the reported exposures to obviously infected other persons, more often resulting in washing-resistant transmission, might have been partially aerogenic. Possible reasons for the overall relatively marginal effect of the water-and-soap intervention have been discussed before [14]. The role of hand hygiene in limiting the transmission of respiratory infections is likely to be much greater in general because the volunteers in this trial had a relatively high standard of hand hygiene already at the start of the trial [14]. This conclusion is based on a standardized questionnaire on daily hand hygiene habits sent to the participants before the onset of and twice during the trial. Several parameters in the intervention arms improved during the trial, and somewhat in the control arm as well [14].

### Effect of interventions on weekly prevalence of reported symptoms of gastrointestinal infection

Gastrointestinal infections occurring without an associated observed exposure were rare, occurring about one per cent of person weeks, and in all trial arms, reported homologous exposure posed a high relative risk of GTI during the same week (jointly for all arms 15.7, 95% CI 12.7 – 19.5, *p* < 0.001) continuing at a moderate level to the following week (3.73, 95% CI 3.07 – 4.5, *p* < 0001). The predictive margins of GTI prevalence in the soap-and-water arm were significantly lower than those of the controls concerning infections occurring during the same and the week following an observed exposure, as well as in association with the weeks when no exposure was recorded (Table 5). Most of the corresponding figures in the alcohol-rub arm were also lower than those in the control arm but none of these differences was statistically significant. Again, this pattern of results, with minor variation in the precise numbers, was obtained when the data was analyzed using different statistical models (data not shown).

**Table 5:** Reported-exposure –dependent efficacy of interventions on weekly prevalence of reported symptoms of gastrointestinal infection (GTI)

Weeks with or without reported exposure Arm and number of weeks	Symptoms reported for					
	Same week			Following week		
	Number and proportion^a^	Predictive margin (CI^b^)	Risk ratio^c^ (CI^b^)	Number and proportion^a^	Predictive margin (CI^b^)	Risk ratio (CI^b^)
Exposure reported						
Controls *n* = 767	159/0.207	0.164 (0.129, 0.209)	NR	67/0.087	0.061 (0.048, 0.079)	NR
Soap-and-water *n* = 937	159/0.170	0.206 (0.166, 0.257)	1.256^d^ (0.878, 1.801)	51/0.054	0.047 (0.036, 0.059)	0.761^e^ (0.537, 1.060)
Alcohol rub *n* = 806	143/0.177	0.186 (0.146, 0.239)	1.130^f^ (0.781, 1.655)	63/0.078	0.056 (0.043, 0.072)	0.904^g^ (0.634, 1.275)
Exposure not reported						
Controls *n* = 10877	154/0.014	0.015 (0.012, 0.018)	NR	226/0.021	0.021 (0.017, 0.027)	NR
Soap-and-water *n *= 14107	123/0.009	0.009 (0.007, 0.011)	0.592^h^ (0.412, 0.847)	210/0.015	0.016 (0.013, 0.020)	0.761^i^ (0.537, 1.060)
Alcohol rub *n* = 11180	131/0.012	0.012 (0.009, 0.015)	0.792^j^ (0.553, 1.145)	193/0.017	0.018 (0.017, 0.027)	0.904^k^ (0.634, 1.275)

*NR* not relevant

^a^Number of weeks with reported RTI symptoms representing indicated proportion out of weeks with reported exposure

^b^CI, 95% credible interval of predictive margins

^c^Risk ratio is the ratio of predictive margin in the intervention arm to that of the control arm

^defghijk^Probabilities that the risk ratio is less than 1.0; ^d^, 0.10; ^e^, 0.95; ^f^, 0.26; ^g^, 0.72; ^h^, 1.0; ^i^, 0.95; ^j^, 0.90; ^k^, 0.72

Note! e = i and g = k; no statistically significant interaction was found in the following week data between arm and exposure. Thus, a single estimate was calculated for the entire arm

The efficacy of the water-and-soap wash containing program thus appeared to be slightly better for prevention of GTI than RTI. This might be considered a conclusion at variance with our previous publication [14]. There, the data was analyzed by straightforward inter-arm comparison of the designated episodes ignoring the potential effects of covariates, and in the conclusions we emphasized the efficacy of soap-and-water washing in the prevention of especially respiratory infections. This notion was based on the fact that we had divided the study period in two parts, weekly reports collected before the 2009 influenza pandemic and those of the remaining time. The strongest effect “before the pandemic” indeed was a reduction of the RTI episodes. The influenza pandemic triggered an intense national hand washing campaign, and no differences were seen between the two interventions and the control arm in the subsequent weekly reports [14]. In the current analysis, however, we avoided the post-hoc division of the study period and included in the model homologous exposure and several covariates. This may have caused the difference in the results. A relatively better efficacy against GTI than RTI is not surprising as such as hand contamination is likely to have a definite role in GTI transmission. Our statistical model also included the seasonality aspect. The 16 months trial lasted from winter 2009 to spring 2010, and the occurrence of both RTI and GTI varied expectedly according to the season. However, calendar-month-wise adjustment of the prevalence did not change the results.

### Additional comments

Weaknesses and limitation of this long-lasting open intervention trial where the collection of data on own infections was based on electronic self-reporting only were discussed previously [14]. The data on reported exposures included in the statistical analysis in this report as a covariate, are likewise based on self-reporting. The exposure data collected was recorded on weekly basis. This was a major reason for the fact that we carried out the current analysis on the efficacy of the interventions by defining the endpoints as weekly prevalence of the RTI or GTI symptoms. This is at variance with the conventional ‘distinct episodes’ –based assessment of interventions, and is likely to cause a slight increase in the numbers of events because some episodes, as defined by successive days with symptoms, will continue over a weekend. However, periods of successive days with RTI or GTI symptoms may, in true life, not always be distinct singular episodes, caused by a defined pathogen. Accumulating evidence indicates that at a given time point, a nasopharyngeal swab specimen may contain two or more different pathogenic viruses [see, for instance ref. [19]], most likely without a temporal coordination of the course of infection. Thus, a continuum of days with symptoms may in fact be a sum effect of two or more partially overlapping infection episodes.

The incubation period of many respiratory and gastrointestinal viral infections is only 1 to 2 days [20]. -Hence, disease symptoms in the reporter recorded for the same week with a homologous exposure, if having any cause-effect relationship to the latter, may be either a consequence or the source of the disease in the contact person. An infection recorded for the week following an observed exposure does not have this bias in a potential cause-effect consideration but may, of course, be unrelated. One can also speculate that although a several days interval between an exposure and an onset of infection might reduce the likelihood of the exposure being an immediate source of the infection, the infection could represent a second round of infections following the exposure. A further weakness in the exposure data is the lack of quantitation. In our data, for instance, a week with reported exposure to RTI may mean a single event during the weak with somebody coughing nearby, or daily physical contacts with a child suffering from a severe cold. It is obvious that the risks of contracting an infection from these two extremes of reported exposure are different.

Potential reasons for the observed absence of protection from infection episodes in the alcohol hand rub intervention arm, in contrast to some earlier studies [21, 22], were also discussed previously [14]. Apart from differences in the chemical composition of the rub, the potential reasons include the relative resistance of the major causative agents, rhinoviruses in RTI and noroviruses in GTI to a brief exposure to alcohol, as implemented in the hand-rub mode of hand cleaning [23, 24].

## Conclusions

The current reanalysis of the STOPFLU data with obvious covariates included in the statistical model confirms the previous conclusion that improved personal hygiene measures consisting of transmission-limiting behavior in coughing, sneezing, and shaking hands, combined with frequent hand washing with soap and water can reduce the occurrence of self-reported acute illnesses in common office work environment. According to our analysis, the effect on GTI is stronger than that towards RTI, possibly due to the partially different transmission routes. In contrast, using a statistical model with the several covariates (personal and cluster specific random effects, self-reported exposure to homologous disease, randomization triplet, and seasonality) included did not change the original conclusion that, in this trial, hand rubbing with alcohol-based disinfectant did not reduce the weekly prevalence of either type of the infection as compared with the control group.

## Additional files

Additional file 1: CONSORT 2010 Flow Diagram. (PDF 212 kb)
Additional file 2: Sensitivíty analysis of predictive margins of weeks with reported symptoms of respiratory tract infections (RTI). (PDF 250 kb)

## Abbreviations

CI: 95% credibility interval
GEE: Generalized estimating equations
GTI: Gastrointestinal tract infection
ReR: Relative risk
RRa: Risk ratio
RTI: Respiratory tract infection
SD: Standard deviation
