# Supplementary figures and images for: Intensified hand-hygiene campaign including soap-and-water wash may prevent acute infections in office workers, as shown by a recognized-exposure -adjusted analysis of a randomized trial
Source: BMC Infect Dis. 2017 Jan 9;17:47. doi: 10.1186/s12879-016-2157-z (PMC5223302; doi:10.1186/s12879-016-2157-z)

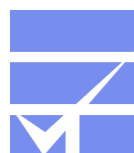

# CONSORT

TRANSPARENT REPORTING of TRIALS

## Additional file 1. CONSORT 2010 Flow Diagram

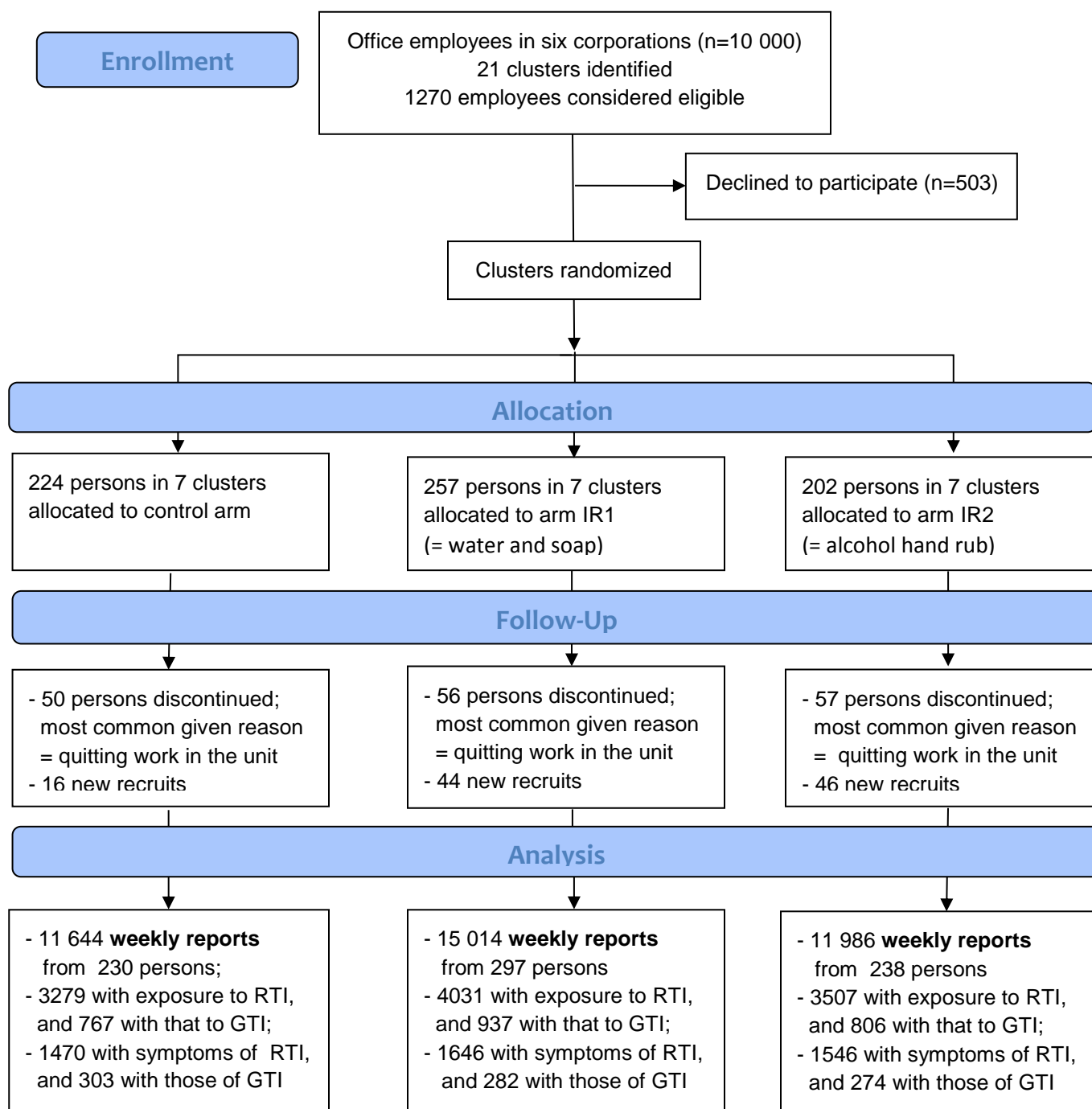

Supplement: Additional file 1: — CONSORT 2010 Flow Diagram. (PDF 212 kb) [file 12879_2016_2157_MOESM1_ESM.pdf]
