# Supplementary material for: Intensified hand-hygiene campaign including soap-and-water wash may prevent acute infections in office workers, as shown by a recognized-exposure -adjusted analysis of a randomized trial
Source: BMC Infect Dis. 2017 Jan 9;17:47. doi: 10.1186/s12879-016-2157-z (PMC5223302; doi:10.1186/s12879-016-2157-z)
Supplement: Additional file 2: — Sensitivíty analysis of predictive margins of weeks with reported symptoms of respiratory tract infections (RTI). (PDF 250 kb) [file 12879_2016_2157_MOESM2_ESM.pdf]

Additional file 2. Sensitivíty analysis of predictive margins of weeks with reported symptoms of respiratory tract infections (RTI).

In the sensitivity analysis with the marginal model (GEE) approach (binary regression with log link) we modelled longitudinal effects using a robust variance estimator and evaluated different sensible autocorrelation structures (1,2). The GEE approach ignored the clustering component because the random effect variance estimates given by Stata 11 in the mixed effect logistic regression model were of the order from 10^{-6} to 10^{-16}. We considered that the effects of clustering to the variance estimates were quite small even if the repeated measurements in the data increased the effective cluster sizes. The "best" autocorrelation structure, chosen by the quasi-information criterion (QIC), was a robust independent. The sensitivity of the results to chosen model (mixed model/GEE) was checked. The predictive margins and their 95% confidence intervals for RTI with GEE modelling using two specific autocorrelation structures (robust independent and exchangeable) are shown in the table (below). When comparing these with our mixed model approach, we see that in overall the results are reasonable well in agreement but the Bayesian mixed model (3) assessed the exposed control and the soap-and-water arm in the same week models to have somewhat larger predictive margins than the GEE model (exhangeable column). Also, there are no systematic differences in the lengths of 95% the CI:s.

Arm	Predictive margins and 95%predictive intervals (second lines) obtained in indicated models for RTI symptoms during same week or following week in relation to reported exposure											
	No exposure reported for index week						Homologous exposure reported for index week					
	Robust exchangeable		Mixed effect bayes model/ exchangeable autocorrelation		Robust independent		Robust exchangeable auto-correlation exposed		Mixed effect Bayes model / exchangeable autocorrelation		Robust independent	
	Same week	Following week	Same week	Following week	Same week	Following week	Same week	Following week	Same week	Following week	Same week	Following week
Control	0.063<br>0.052, 0.074	0.083<br>0.073, 0.092	0.063<br>0.055, 0.072	0.084<br>0.074 0.096	0.059<br>0.050, 0.069	0.078<br>0.069, 0.087	0.275<br>0.244, 0.305	0.216<br>0.194, 0.243	0.292<br>0.258, 0.329	0.216<br>0.192, 0.246	0.286<br>0.251, 0.320	0.239<br>0.211, 0.268
Soap-and-water	0.048<br>0.040, 0.056	0.077<br>0.069, 0.086	0.042<br>0.037, 0.048)	0.073<br>0.065 0.081	0.046<br>0.038, 0.053	0.070<br>0.062, 0.077	0.283<br>0.252, 0.315	0.204<br>0.181, 0.226	0.306<br>0.276, 0.339	0.188<br>0.168, 0.208	0.279<br>0.246, 0.312)	0.213<br>0.189, 0.238
Alcohol-rub	0.060<br>0.049, 0.071	0.082<br>0.072, 0.092	0.062<br>0.054, 0.071	0.081<br>0.072, 0.091	0.061<br>0.051, 0.071	0.077<br>0.067, 0.087	0.280<br>0.248, 0.311	0.216<br>0.191, 0.241	0.281<br>0.245, 0.317	0.207<br>0.185, 0.235	0.281<br>0.245, 0.316	0.237<br>0.209, 0.265

References:

1. Campbell MJ. Cluster Randomized trials. In: Ahrens W, Pigeot I, editors. Handbook of Epidemiology. 2nd Edition ed2014. New York; Springer; 2014. p. 389-417.

2.Hosmer DJ, Lemeshow S, Sturdivant S. Applied logistic regression. New Jersey; Wiley; 2013. p. 339-44.

3. Turner RM, Omar RZ, Thompson SG. Bayesian methods of analysis for cluster randomized trials with binary outcome data. Stat Med. 2001 Feb 15;20(3):453-72.
